# Effect of *Moringa oleifera* stem extract on hydrogen peroxide-induced opacity of cultured mouse lens

**DOI:** 10.1186/s12906-019-2555-z

**Published:** 2019-06-21

**Authors:** Lei Qi, Yu Zhou, Weijie Li, Mali Zheng, Ruisheng Zhong, Xin Jin, Yuan Lin

**Affiliations:** 1 Department of Ophthalmology, Xiamen Hospital of Traditional Chinese Medicine, Xiamen, 361005 People’s Republic of China; 2 Department of Basic Medical Science, School of Medicine, Xiamen University, Xiamen, 361102 People’s Republic of China

**Keywords:** *Moringa oleifera*, Lens, Organ culture, Oxidative stress, Antioxidant

## Abstract

**Background:**

*Moringa oleifera,* also known as horseradish tree or drumstick tree, has strong antioxidant properties. In the present study, we investigated the potential effect of *Moringa oleifera* stem extract (MOSE) on cataract formation induced by oxidative stress in cultured mouse lenses.

**Methods:**

Mouse lenses cultured in vitro were pretreated with MOSE (0.5 and 1 mg/mL) for 24 h. Then, 1 mM hydrogen peroxide was added, and mouse lenses were cultured for a further 24 h. The medium was then changed to normal culture medium. After 48 h, lens opacification, reactive oxygen species (ROS) generation, reduced glutathione (GSH) content, and activities of superoxide dismutase (SOD) and catalase (CAT) were measured in lens tissues. In addition, the protein expression of peroxisome proliferator-activated receptor alpha (PPARα), a nuclear receptor with potential benefits to improve vision-threatening eye diseases, was assayed.

**Results:**

MOSE (1 mg/mL) alleviated lens opacification, reduced ROS generation, increased GSH content, and elevated SOD and CAT activities in cultured lenses. Moreover, MOSE upregulated the expressions of SOD, CAT, and PPARα.

**Conclusions:**

This study showed that MOSE alleviates oxidative stress-induced cataract formation, and the mechanism of the effect is mainly related to its improvement of the endogenous antioxidant system in the lens.

**Electronic supplementary material:**

The online version of this article (10.1186/s12906-019-2555-z) contains supplementary material, which is available to authorized users.

## Background

The lens has a transparent, elastic avascular refractive organization and plays an important role in visual formation. Cataracts are characterized by gradual accumulation of cloudy deposits on the ocular lens and have been a leading cause of visual impairment and blindness worldwide for centuries. Although modern cataract surgery is safe and effective, there are still many problems, such as high costs, loss of normal functions of postoperative eyes, and a high incidence of after-cataract. As the aging population increases, cataracts have become an increasingly serious issue [1, 2]. Thus, there is a great demand for safe, effective, and inexpensive agents to prevent or delay the onset of cataracts.

Cataracts are multifactorial eye diseases associated with several risk factors such as aging, diabetes, exposure to sunlight, and hypertension. However, oxidative stress caused by reactive oxygen species (ROS) has long been regarded as the major mechanism by which cells are damaged and cataracts are formed [3–6]. Under physiological conditions, lenses can compensate for a mild degree of oxidant stress and remove oxidative damaged molecules by elevating endogenous antioxidants such as reduced glutathione (GSH) and activating anti-oxidant enzymes, such as superoxide dismutase (SOD) and catalase (CAT), which play important roles in protecting the lens against oxidative stress. However, in some cases such as aging, ROS production is excessive or the ability of the lens to scavenge ROS decreases, and oxidative stress injuries may occur and then cataracts are formed [3, 4].

Because of the major role of oxidative stress in cataract formation, natural antioxidants with high activity and few side effects have attracted increasing attention to delay the onset or progression of cataracts [7–10]. *Moringa oleifera*, also known as horseradish tree or drumstick tree, belongs to the *Moringaceae* family and has been used in nutritious foods and traditional medicines for the treatment of various diseases such as rheumatism, inflammation, and diabetes in many Asian countries [11]. Particularly, *Moringa oleifera* is one of the best known and most widely distributed species that is rich in natural antioxidants [12, 13]. *Moringa oleifera* leaf extracts was reported to inhibit the ROS formation induced by H_2_O_2_ and enhanced the activities and mRNA expressions of SOD and CAT in KB cells [14] and in HEK-293 Cells [15]. Recently the *Moringa oleifera* leaf extract was reported to protect yeast cells against oxidative stress induced by cadmium and H_2_O_2_ through the reduction of intracellular ROS levels [16]. Regular intake of *Moringa oleifera* leaves through diet decreased the lipid per oxidation and increase the SOD and CAT activities in a diabetes-induced oxidative stress model in rats [17]. *Moringa oleifera* seed extract can inhibit the ROS formation induced by high fat diet in mice [18]. *Moringa oleifera* root extract attenuated beryllium-induced oxidative stress in rats [19]. All these significant antioxidant activities of *Moringa oleifera* from both in vitro as well as in vivo studies suggest that *Moringa oliefera* may inhibit the cataract formation induced by oxidative stress. Although some studies have reported that the *Moringa oleifera* leaf extract has potential inhibitory effects on high sugar-induced cataract in goat lens in vitro [20] and selenite-induced cataract in rat pups [21], no study have actually been conducted on the protective effects of *Moringa oleifera* on oxidative stress induced cataract. In addition, compared with the traditional uses of the leaves, flowers, and seeds of *Moringa oleifera*, its stem is not often consumed, and the stem may even be considered as an agricultural by-product. However, *Moringa oleifera* stem is very abundant and inexpensive. Thus, any health benefit from it may reach a large part of the population. Therefore, it is worthwhile to investigate the potential effects of *Moringa oleifera* stem on delaying the onset or progression of cataracts induced by oxidative stress. In addition, PPARs (including three isoforms: α, γ, and β/δ) are ligand-activated transcription factors of the nuclear hormone receptor and play key roles in maintaining glucose and lipid homeostasis by modulating gene expression. Recent studies indicate that PPARs have potential benefits to improve or prevent various vision-threatening eye diseases such as diabetic retinopathy, glaucoma, and diabetic macular edema [22–24]. Therefore, in this research, we also evaluate the effect of *Moringa oleifera* stem on the expression of PPARs.

Lens organ cultures provide a simple and effective platform to screen for candidate compounds that protect against cataract formation [25, 26]. Hydrogen peroxide (H_2_O_2_) is the main intracellular ROS in the aqueous humor, which causes cataract development [27, 28], and is often used to induce cataract formation in vitro [8]. Therefore, in the present study, we focused on the protective effect of *Moringa oleifera* stem extract (MOSE) against cataract formation and explored its underlying mechanism using a cataract formation model induced by H_2_O_2_ in lens organ culture. Luteolin is a flavonoid present in the leaves and stems of many plants and some reports indicate that luteolin exerts protective effects on selenite [29, 30] and STZ [31]-induced cataracts. So luteolin was used as a reference for its established antioxidant property in our research.

## Methods

### Animals

Experiments were performed using male BALB/c mice weighing 20–22 g (Certificate No. SCXK 2012–0002; Shanghai SLAC Laboratory Animal Co. Ltd., Shanghai, China). Mice were housed under a controlled temperature (22 ± 1 °C) with a 12 h light/dark cycle, and allowed free access to food and water. All experimental protocols described in the present study were approved by the Animal Care and Use Committee of Xiamen University (LACUC: XMULAC20150077). All procedures for the animal study were conducted in accordance with ARRIVE guidelines, and every effort was made to alleviate the suffering of the animals.

### Reagents

Medium 199 (M-199), fetal bovine serum (FBS), and an antibiotic solution were purchased from Gibco (Grand Island, NY, USA). 2,2-Diphenyl-1-1-picrylhydrazyl (DPPH), rutin, and luteolin were purchased from Sigma-Aldrich (St. Louis, MO, USA). 2′, 7′-Dichlorofluorescein diacetate (DCFH-DA) and radioimmunoprecipitation assay (RIPA) lysis buffer were purchased from Solarbio (Beijing, China). Other materials used are specified in detail in the following sections.

### Preparation of MOSE

Dry *Moringa oleifera* stems were purchased from Xiamen Jinzhu Ecological Agriculture Co. Ltd. (Xiamen, Fujian, China). Plant identification was done by Dr. Chenqin, an expert from the School of Pharmaceutical Science of the Xiamen University (Xiamen, China). The voucher specimen (No. 20140002) was kept at the Key Laboratory of Chiral Drugs, Medical College, Xiamen University, Xiamen, China. The dry *Moringa oleifera* stems were then powdered and extracted with 70% ethanol at 85 °C for 2 h. Then, the supernatant was filtered using Whatman filter paper and vacuum evaporated to obtain the ethanol MOSE. The extracts were freeze dried into powder form for storage. For experimental use, the freeze-dried powder of MOSE was freshly diluted with M-199 and then filtered through a 0.22 μM microfiltration membrane.

### Determination of the total flavonoid content in MOSE

The total flavonoid content in MOSE was measured using a NaNO_2_-Al (NO_3_)_3_-NaOH colorimetric assay [32]. Briefly, 10 mL MOSE solution (0.1 mg/mL, diluted with 100% ethanol) was mixed with 1 mL of 5% NaNO_2_, and then 1 mL of 10% Al(NO_3_)_3_ was added. After 5 min, 5 mL of 1 M NaOH was added to the mixture. The volume was increased to 25 mL with 60% ethanol, and the mixture was allowed to rest for 15 min. Absorbance was measured at 510 nm. All determinations were performed in triplicate. The total flavonoid content was expressed as mg of rutin equivalents per g of dried MOSE.

### Assay of the DPPH radical scavenging capacity

The effects of MOSE and luteolin on DPPH scavenging were measured according to a previously reported method [33]. A DPPH radical solution was prepared by dissolving 2 mg DPPH in 50 mL of 70% EtOH. MOSE or luteolin was also dissolved in 70% EtOH at various concentrations. The DPPH radical solution (225 μL) was mixed with 75 μL of each sample in a 96-well microplate. An equal volume of EtOH was added to the control well. Absorbance at 517 nm was measured after 30 min of reaction at room temperature in the dark using a microplate reader. Lower absorbance of the reaction mixture indicated a higher DPPH free radical scavenging activity. The percentage of DPPH radical inhibition was calculated as follows:

DPPH radical inhibition (%) = 100% × [(A – B) / A].

Where A is the absorbance value of the control reaction (containing DPPH solution only) and B is the absorbance value of the test reaction (containing the DPPH solution and sample). The antioxidant activity of the compound was expressed as IC_50_ and is defined as the concentration (l g/mL) of compound that inhibited the formation of DPPH radicals by 50%.

### Lens organ culture

The lens organ culture was prepared according to previous reports with some modifications [25, 34, 35]. First, mice were brought to the carbon dioxide (CO_2_) euthanasia apparatus and then exposed to CO_2_ until complete cessation of breathing was observed. The mice were then be decapitated and the lenses were isolated through the posterior approach from the eyes and transferred to 6-well plates containing 4 mL M-199 medium with 1% penicillin-streptomycin and 2% FBS per well. Then, the lenses were incubated at 37 °C in a 5% CO_2_ incubator. After 24 h, each lens was observed under an anatomical microscope (Leica S6D; Leica Microsystems, Wetzlar, Germany), and transparent lenses were selected for further experiments. The selected lenses were divided into the following groups: normal control group (lenses cultured in normal medium without H_2_O_2_ exposure); MOSE-treated control group (lenses cultured in medium with 1 mg/mL MOSE without H_2_O_2_ exposure); vehicle control group (lenses cultured in normal medium before H_2_O_2_ exposure); MOSE (0.5 mg/mL)-treated group (lenses cultured in medium with 0.5 mg/mL MOSE before H_2_O_2_ exposure); MOSE (1 mg/mL)-treated group (lenses cultured in medium with 1 mg/mL MOSE before H_2_O_2_ exposure); luteolin (0.05 mg/mL)-treated group (lenses cultured in medium with 0.05 mg/mL luteolin before H_2_O_2_ exposure).

The lenses were incubated in the medium for 24 h and then treated with or without 1 mM H_2_O_2_ for 24 h, followed by incubation in fresh medium for another 48 h. At the end of the experiments, each lens was examined under the anatomical microscope for morphological changes and then removed from the culture dish, carefully blotted on wet filter paper, weighed, and then immediately frozen for subsequent analysis.

### Measurement of lens opacification

The opacity of lens in each group (*n* = 6 per group) was examined under the anatomical microscope equipped with a charge-coupled device camera. The mean gray value of each lens was measured according to a previous report [36] using ImageJ software (Wayne Rasband National Institutes of Health, USA). The results were expressed as the fold change of the average gray value of the lens from that in the normal control group.

### Assessment of ROS generation in lenses

Direct evidence of intracellular oxidation was observed in lens homogenates using the oxidant sensitive probe DCFH-DA, according to the method of other’s reports [37–40] with a slight modification. The DCFH-DA fluorescent probe is oxidized by ROS to produce DCF that is highly fluorescent at 530 nm. At the end of the experiments, the lenses (*n* = 6 per group) were homogenized in a glass homogenizer with 0.9% saline at a ratio of 1:9. To measure ROS generation, the homogenates (100 μL) of each sample were mixed with 100 μL DCFH-DA (20 μM) in a 96-well microplate, and then incubated at 37 °C in the dark for 30 min. The homogenates were centrifuged at 3000×*g* for 15 min at 4 °C, and the fluorescence of the supernatants was measured using a spectrofluorometer (488 nm excitation and 520 nm emission; Varioskan, Thermo, USA). The result was calculated as the fluorescence intensity per mg of protein and expressed as the fold change of fluorescence intensity from the normal control group.

### Assays of GSH content and activities of anti-oxidative enzymes (SOD and CAT) in lens

After experiments, the lenses were washed with cold 0.9% saline, dried with filter paper, and weighed. Then, the lenses (*n* = 6 per group) were homogenized in a glass homogenizer with 0.9% saline at a ratio of 1:9. The homogenates were centrifuged at 3000×*g* for 15 min at 4 °C, and the supernatants were collected for assays. GSH content and total SOD and CAT activities were measured using specific assay kits (Nanjing Jiancheng Bioengineering Institute, Nanjing, China), according to the manufacturer’s instructions. The protein content of the supernatant was determined using a BCA kit (Applygen Technologies Inc., Beijing, China). For all assays, the activity was calculated as the fold change from the control.

### Western blot analysis

At the end of experiments, the lenses (*n* = 3 per group) were washed with PBS and lysed in RIPA buffer containing protease inhibitors (Aidlab Biotechnologies, Beijing, China) for 30 min on ice. The lens lysates were centrifuged at 12,000×*g* for 20 min at 4 °C, and the proteins were quantified using the BCA kit. Protein samples (80 μg) were separated by 10% sodium dodecylsulfate-polyacrylamide gel electrophoresis and transferred to polyvinylidene fluoride membranes (Millipore, Billerica MA, USA). The membranes were blocked with 5% fat-free dry milk for 2 h and then incubated with a rabbit polyclonal antibody against SOD (1:300, R&D Systems, Minneapolis, MN, USA), CAT (1:300, Abcam, Cambridge, MA, USA), peroxisome proliferator-activated receptor alpha (PPARα) (1:500, Abcam, Cambridge, MA, USA), or GAPDH (1:1000, R&D Systems MN, USA) at 4 °C overnight. Then, the membranes were incubated with horseradish peroxidase-conjugated anti-rabbit IgG (1:1000, Cell Signaling Technology Inc., USA) for 2 h. Finally, the protein bands were developed using enhanced chemiluminescence reagents (Millipore). Images were obtained using a Kodak Image Station 4000R (Eastman Kodak Co., Rochester, NY, USA) and analyzed using Kodak Image Software. The optical densities of specific immunopositive bands were normalized to the GAPDH band in the same sample.

### Statistical analyses

Each experiment was performed at least three times. The results are expressed as means ± standard error of the mean (SEM). Statistical analyses were performed by one-way analysis of variance, followed by Tukey’s post-hoc test using Prism 5 software for Windows (GraphPad Software Inc., San Diego, CA, USA). Values of *P* < 0.05 were considered as statistically significant.

## Results

### Total flavonoid content in MOSE

The total flavonoid content was measured by a NaNO2-Al (NO3)3-NaOH colorimetric assay. The flavonoid content in MOSE was 169.7 ± 3.015 mg rutin equivalents/g MOSE dry weight, indicating that 1 g MOSE is equivalent to 169.7 mg rutin.

### DPPH-scavenging capacity

**Fig. 1:**
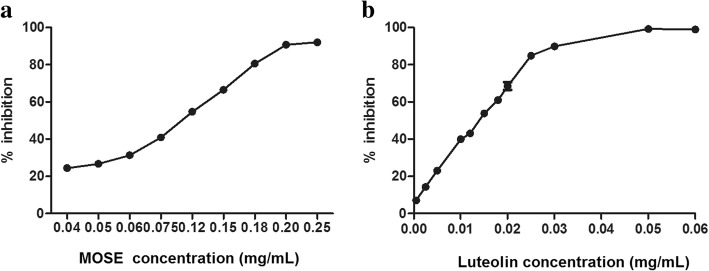
DPPH radical scavenging assay of MOSE (**a**) and luteolin (**b**). The results are expressed as percentage inhibition of DPPH radical formation. Data are expressed as the mean ± SEM of three separate experiments

To determine the effect of MOSE and luteolin on radical scavenging, we measured their effects on scavenging DPPH radicals. Both MOSE and luteolin significantly reduced DPPH radicals in a dose-dependent manner (*P* < 0.05 vs. control group, Fig. 1a). The IC_50_ of DPPH radical scavenging was 0.105 ± 0.0004 mg/mL for MOSE (Fig. 1a) and 0.014 ± 0.0007 mg/mL for luteolin (Fig. 1b). Therefore, the free radical scavenging activity of 1 mg MOSE was approximately equivalent to that of 0.13 mg luteolin.

### Effects of MOSE and luteolin on lens opacity

**Fig. 2:**
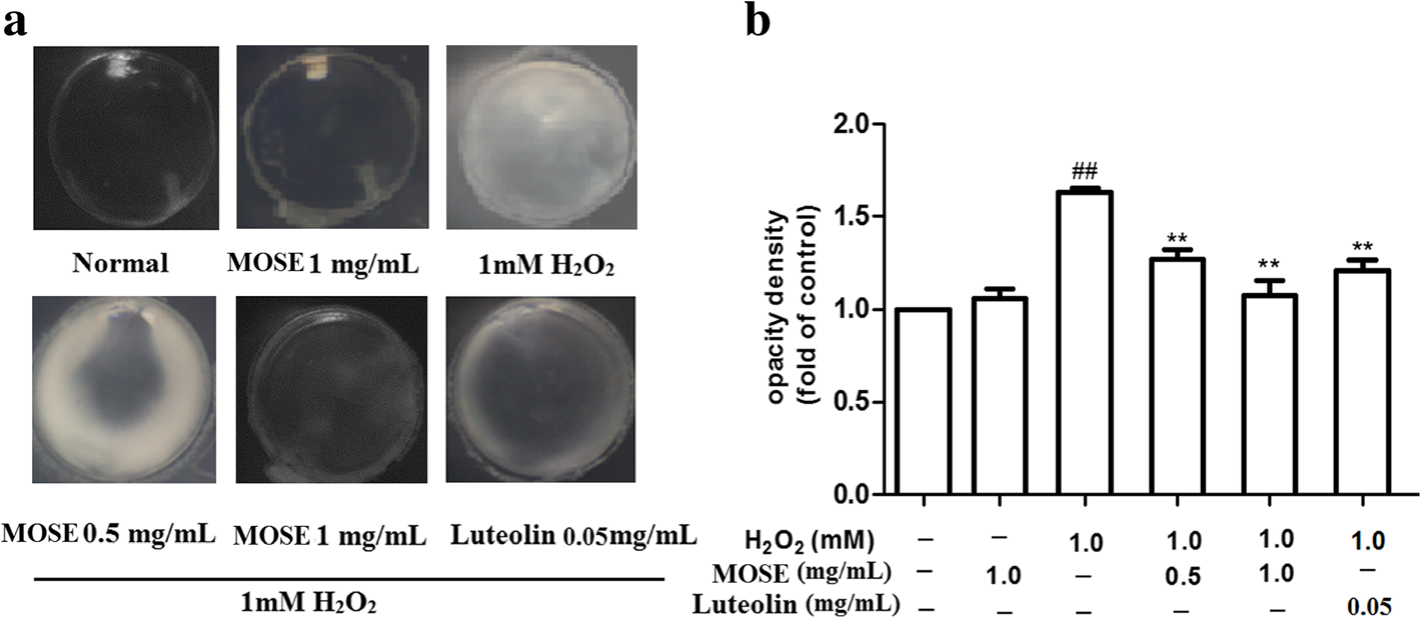
Effects of MOSE and luteolin on lens opacity in H_2_O_2_-induced cataract. Lenses were pretreated with MOSE (0.5 and 1.0 mg/mL) or luteolin (0.05 and 0.1 mg/mL) for 24 h, followed by incubation with H_2_O_2_ (1 mM) for another 24 h and then recovered in fresh medium for 48 h. **a** Representative photograph showing the lens with different treatment. **b** Quantificaton of lens opacity density. Data are expressed as the mean ± SEM (*n* = 6); ^##^*P* < 0.01, compared with the normal control group; **P < 0.01 compared with the H_2_O_2_ treatment group

To examine the effects of MOSE and luteolin on lens opacity, the change in lens opacity induced by H_2_O_2_ was observed under a stereomicroscope. All lenses in normal control and MOSE pretreatment control groups were transparent, but 1 mM H_2_O_2_ remarkably induced dense cortical vacuolization and opacification (Fig.2 a). After pre-treatment with MOSE (0.5 and 1.0 mg/mL), the H_2_O_2_-induced lens opacity levels were reduced remarkably (Fig. 2b). In our preliminary experiment, we found that luteolin as a control also reduced lens opacity, and the effect of luteolin at 0.05 mg/mL was better than that at 0.1 mg/mL (Additional file 2: Figure S1). Therefore, 0.05 mg/mL luteolin was used in the formal experiments.

### Effects of MOSE and luteolin on ROS accumulation, GSH content, and SOD/CAT activities in lens

**Fig. 3:**
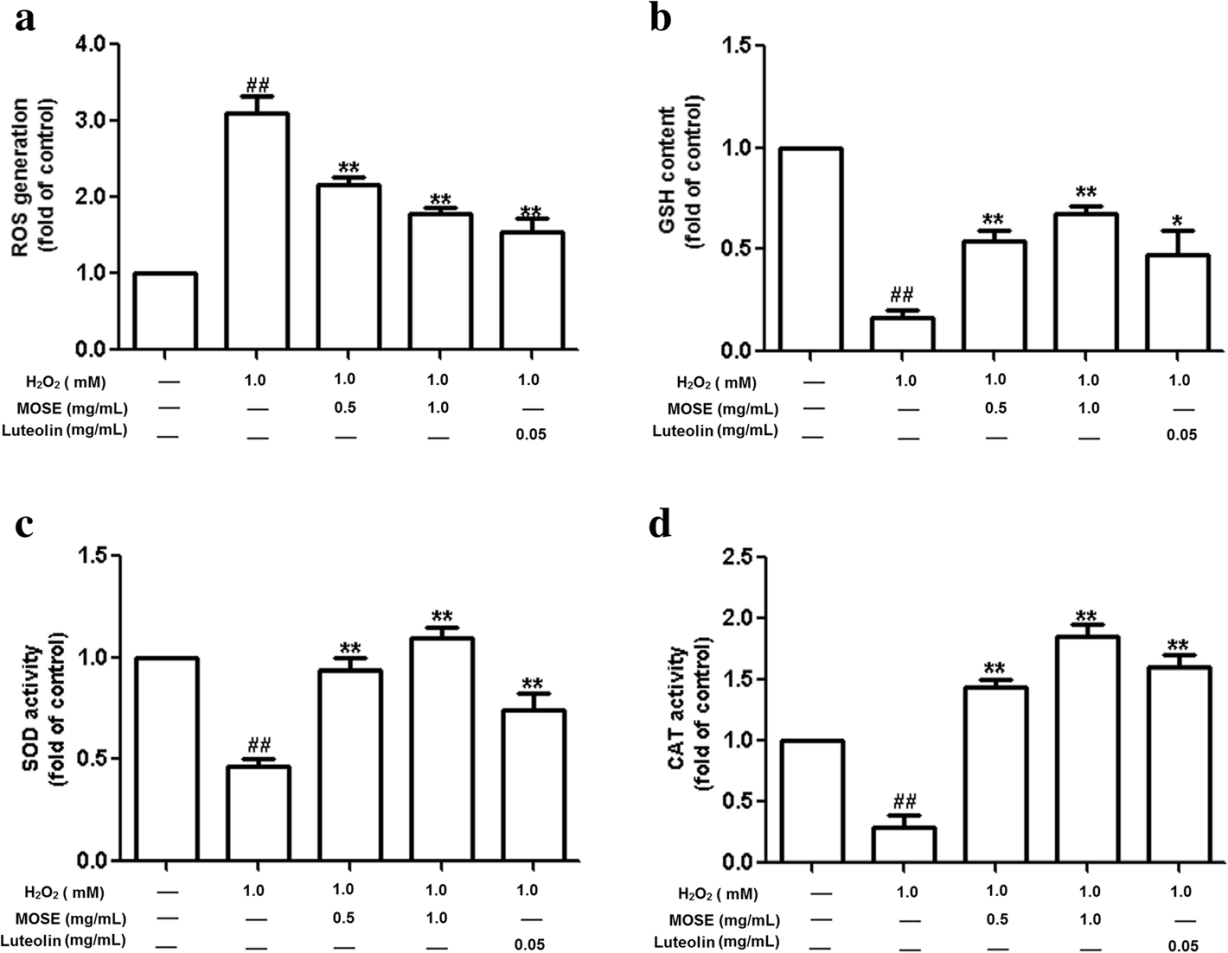
Effects of MOSE and luteolin on ROS production and SOD, CAT activities in lenses. The fluorescent probe DCFH-DA was used to detect the ROS level in lenses pre-treated with MOSE (0.5 and 1.0 mg/mL) or luteolin (0.05 mg/mL) before H_2_O_2_ exposure. The fluorescent intensities are summarized in **a**. GSH content (**b**), and SOD (**c**) and CAT (**d**) activities were measured in lenses pretreated with MES or luteolin before H_2_O_2_ exposure. Data are expressed as the mean ± SEM (n = 6); ^##^P < 0.01, compared with the normal control group; *P < 0.05, **P < 0.01, compared with the H_2_O_2_ treatment group

To investigate whether MOSE prevents H_2_O_2_-induced ROS generation, ROS levels in lenses were measured using the fluorescent probe DCFH-DA. The basal level of ROS in mouse lens was 16.41 ± 1.018 (fluorescence intensity/mg protein), ROS level in lens tissue increased to 50.88 ± 3.66 (fluorescence intensity/mg protein), about 3 folds to basal level, after exposure to 1 mM H_2_O_2_ for 24 h (Additional file 1: Table S1 and Fig. 3a), and MOSE (0.5 and 1 mg/mL) significantly decreased H_2_O_2_-induced ROS production (*P* < 0.01, Fig. 3a). As a control, luteolin also reduced the production of ROS in lenses. There was no obvious difference between MOSE and luteolin for inhibiton of ROS accumulation in lenses (Fig. 3a).

To investigate the mechanisms underlying the antioxidant effect of MOSE, we observed the effect of MOSE on GSH content and the activities of some antioxidant enzymes that participate in ROS degradation. H_2_O_2_ (1 mM) markedly decreased GSH content and reduced the activities of SOD and CAT. MOSE (0.5 and 1 mg/mL) significantly increased GSH content and SOD activities in lenses after H_2_O_2_ treatment (*P* < 0.01, Fig. 3b, c). Furthermore, MOSE remarkably increased the activity of CAT in lenses by almost 2-fold compared with the control group (Fig. 3d). The effects of MOSE on GSH content and antioxidant enzyme activities were much more potent than those of luteolin in lenses.

### Effects of MOSE and luteolin on expressions of SOD, CAT, and PPARα

**Fig. 4:**
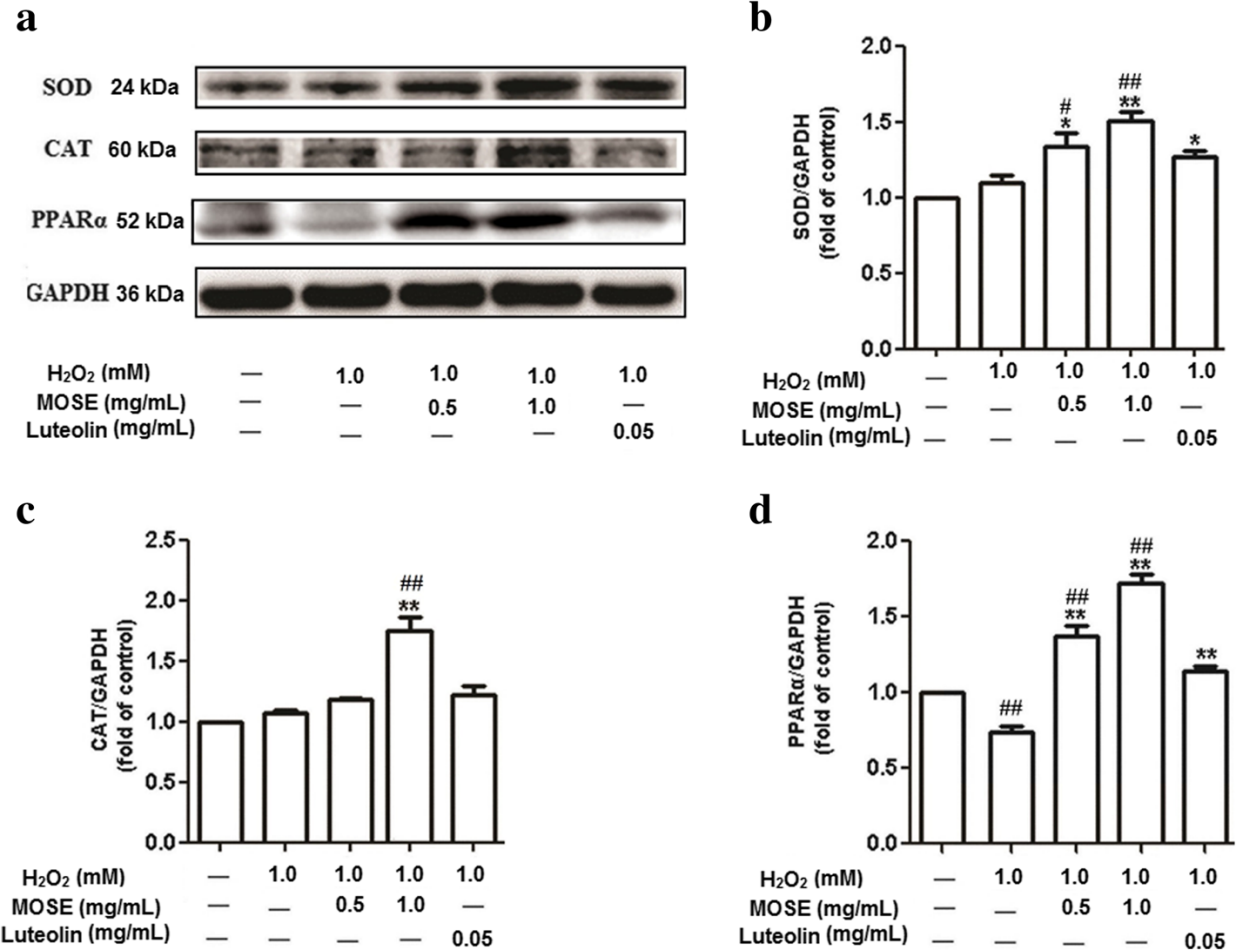
Effects of MOSE and luteolin on expressions of SOD, CAT and PPARα. Lenses were pretreated with MOSE (0.5 and 1.0 mg/mL) or luteolin (0.05 mg/ mL) for 24 h, followed by incubation with H_2_O_2_ (1 mM) for another 24 h, and then recovered in fresh medium for 48 h. Then, protein expressions were  determined by  western blot (**a**). Quantification of western blot  for SOD (**b**), CAT (**c**), and PPARα (**d**). Data are expressed as the mean ± SEM (*n* = 3); ^#^P < 0.05 and ^##^P < 0.01, compared with the normal control group; *P < 0.05 and **P < 0.01, compared with the H_2_O_2_ treatment group

To investigate the molecular mechanisms underlying the antioxidant activity of MOSE, protein expression of SOD and CAT was determined. Pre-treatment with MOSE (1 mg/mL) significantly increased the expression of SOD and CAT (*P* < 0.01, Fig. 4a, b, c; Additional file 3: Figure S2). Luteolin (0.05 mg/mL) also increased the expression of SOD, but had no effect on expression of CAT. PPARα is a ligand-activated transcription factor that plays a key role in modulating the redox balance [41]. Therefore, we examined the effect of MOSE on PPARα expression in lenses. MOSE (0.5 and 1 mg/mL) increased PPARα expression in lenses after oxidative stress (P < 0.01, Fig. 4d; Additional file 3: Figure S2). Luteolin restored the protein expression of PPARα, but did not increase its expression (Fig. 4d).

## Discussion

Oxidative stress plays a major role in cataract formation, and H_2_O_2_ is one of the major oxidants that appears to contribute to cataract formation [42, 43]. In this study, lens organ cultures were used to observe the protective effect of MOSE against lens opacity induced by H_2_O_2_. Although harvesting the lens from the mouse eye without inducing mechanical injury is technically challenging, lens organ culture is a simple and effective method to evaluate the effect of candidate compounds on cataract formation [25, 26]. Our study showed that whole lenses isolated from mouse eyes remained transparent in M-199 medium with 2% FBS, and all lenses without protection became opaque after exposure to 1 mM H_2_O_2_ for 24 h. MOSE (0.5 and 1.0 mg/mL) reduced the opacity of the lens, and 1 mg/mL MOSE was more effective than 0.5 mg/mL MOSE. Previous reports indicate that *Moringa oliefera* has potential inhibitory effects on selenite-induced cataract in rat pups [21] and high sugar-induced cataract in goat lens in vitro [20]. However, our study shows for the first time that the ethanol extract of *Moringa oleifera* stem, an agricultural by-product of *Moringa oliefera,* can also effectively prevent cataract formation induced by H_2_O_2_, a major intracellular ROS, in lens organ culture.

Free radicals are thought to increase the risk of cataract. Our results indicate that MOSE reduces the levels of DPPH free radicals in a cell-free system and inhibits the production of ROS in lenses. However, we also found that, although the effect of luteolin on scavenging free radicals was much better than that of MOSE, the inhibitory effect of MOSE on cataract formation was better than that of luteolin. This result suggests that quenching free radicals directly may not be the main reason for MOSE-mediated inhibition of cataract formation. MOSE may also prevent cataract formation by regulating cellular antioxidant systems. Our study showed that MOSE significantly restored the level of GSH in cultured lenses. There are non-enzymatic and enzymatic endogenous antioxidant systems in human lenses. GSH is an endogenous antioxidant with high content in lenses [44, 45]. It eliminates superoxide anions, H_2_O_2_, and other oxygen free radicals. In addition, GSH maintains the reduced state of lens proteins, prevents protein denaturation, and plays an important role in the maintenance of lens transparency [46, 47].

Furthermore, our study indicates that MOSE upregulates the activities and expressions of SOD and CAT in lenses after H_2_O_2_ exposure. SOD catalyzes the conversion of superoxide anions to H_2_O_2_ plus dioxygen. CAT catalyzes the conversion of H_2_O_2_ to water and oxygen, preventing oxidative damage [48, 49]. SOD and CAT belong to the antioxidant system that reduces free radicals to less toxic states and play an important role against oxidative stress injury. These results are consistent with another report in which leaf extracts of *Moringa oliefera* increased the mRNA expression levels of some antioxidant enzymes in HEK-293 cells treated with H_2_O_2_ [15]. As a control, the effects of luteolin on endogenous antioxidant defense were not as potent as those of MOSE. A possible mechanism may be that *Moringa oleifera* contains more types of antioxidant compounds, such as ascorbic acid, flavonoids, phenolics, and carotenoids [12], which may confer more potent protection by activating the cellular antioxidant system, thereby providing better protection. Therefore, it appears that MOSE may be a more attractive agent to prevent cataract formation.

Another interesting finding of our study was that MOSE markedly increased PPARα expression at the two doses applied to lenses after oxidative stress. PPARs have been reported to improve or prevent various eye diseases [22–24]. Our study also indicated that lenses with high PPARα expression retained transparency after oxidative stress, and that MOSE upregulated PPARα expression in lenses. These results are in line with another report showing that an extract of *Moringa oleifera* seed increased PPARα expression in cardiac tissue and exerted protective effects [50]. Genomic and biochemical analyses suggest that oxidative stress might play a significant role in the toxic effects of PPARδ or PPARγ agonists on lenses [26]. In contrast, PPARα has been regarded to play a key role in modulating the redox balance [41, 51, 52]. PPARα acts as a transcription factor for diverse target genes possessing a PPAR response element (PPRE) in their promoter region. The PPRE, the binding site for PPARα, has been identified in the promoter regions of genes encoding SOD [53] and CAT [54]. Therefore, SOD and CAT are regarded as target enzymes of PPARα. In fact, many total extracts of natural plants, such as the stems of *Cucurbita moschata* and leaves of *Camellia sinensis*, have been reported to modulate PPARα activity [55]. Collectively, these results indicate that PPARα may be involved in the inhibitory effect of MOSE on cataract formation induced by oxidative stress. However, the detailed role of PPARα in the lens requires further investigation. In addition, we only examined the effect of the crude extract of *Moringa oleifera* stem in this study, and specific phytochemicals in MOSE have not been comprehensively evaluated. Further studies are required to identify the active flavonoids in the fraction responsible for the anti-cataract formation effect and PPARα-activating effect in the future.

## Conclusion

In summary, the results of this study show that MOSE significantly delays the progression of H_2_O_2_-induced cataract formation in lens organ culture, and the mechanisms underlying its effect are related to scavenging of free radicals, increasing GSH content, and enhanced activities and expressions of SOD, CAT, and PPARα. *Moringa oleifera* stem, as a kind of natural antioxidant, has extensive sources and no obvious adverse effects. Therefore, the inhibitory effect of MOSE on cataract formation has great clinical interest. It can be used as a potential natural medicine in the prevention or treatment of cataract, especially induced by diabetes.

## Additional files

Additional file 1: **Table S1.** Quantitative analysis of ROS in lens(fluorescence intensity/mg protein, means ± SEM, *n* = 6. (DOCX 19 kb)

Additional file 2: **Figure S1.** Effects of luteolin on lens opacity in H_2_O_2_-induced cataract. Lenses were pretreated with luteolin (0.01, 0.05 and 0.1 mg/mL) for 24 h, followed by incubation with H_2_O_2_ (1 mM) for another 24 h and then recovered in fresh medium for 48 h. Data are expressed as the mean ± SEM (n = 6); ##*P* < 0.01, compared with the normal control group; **P < 0.01 compared with the H_2_O_2_ treatment group. (TIF 584 kb)

Additional file 3: **Figure S2.** The original gel images of Fig. 4a. (TIF 155 kb)

## Data Availability

All data generated or analyzed during this study are included in this published article. More details are available from the corresponding author on reasonable request.

## Abbreviations

CAT: Catalase
DCFH-DA: 2′, 7′-Dichlorofluorescein diacetate
DPPH: 2,2-Diphenyl-1-1-picrylhydrazyl
FBS: Fetal bovine serum
GSH: Reduced glutathione
H_2_O_2_: Hydrogen peroxide
M-199: Medium 199
MOSE: *Moringa oleifera* stem extract
PPARα: Proliferator-activated receptor alpha
RIPA: Radioimmunoprecipitation assay
ROS: Reactive oxygen species
SOD: Superoxide dismutase
