# Supplementary material for: Effect of Moringa oleifera stem extract on hydrogen peroxide-induced opacity of cultured mouse lens
Source: BMC Complement Altern Med. 2019 Jun 21;19:144. doi: 10.1186/s12906-019-2555-z (PMC6588927; doi:10.1186/s12906-019-2555-z)
Supplement: Supplementary file 1 — Table S1. Quantitative analysis of ROS in lens(fluorescence intensity/mg protein, means ± SEM, n = 6. (DOCX 19 kb) [file 12906_2019_2555_MOESM1_ESM.docx]

**Supplemental Table 1.** Quantitative analysis of ROS in lens（fluorescence intensity/mg protein, means ± SEM, n=6）

| Group | Con | Only H_2_O_2_ | MSE(0.5mg/mL) + H_2_O_2_ | MSE(1mg/mL) +H_2_O_2_ | Luteolin(0.05mg/mL) + H_2_O_2_ |
| --- | --- | --- | --- | --- | --- |
| ROS | 16.41±1.01 | 50.88±3.66 | 35.42±1.707 | 29.19±1.4 | 25.38±2.74 |
