# Supplementary figures and images for: Effect of Moringa oleifera stem extract on hydrogen peroxide-induced opacity of cultured mouse lens
Source: BMC Complement Altern Med. 2019 Jun 21;19:144. doi: 10.1186/s12906-019-2555-z (PMC6588927; doi:10.1186/s12906-019-2555-z)

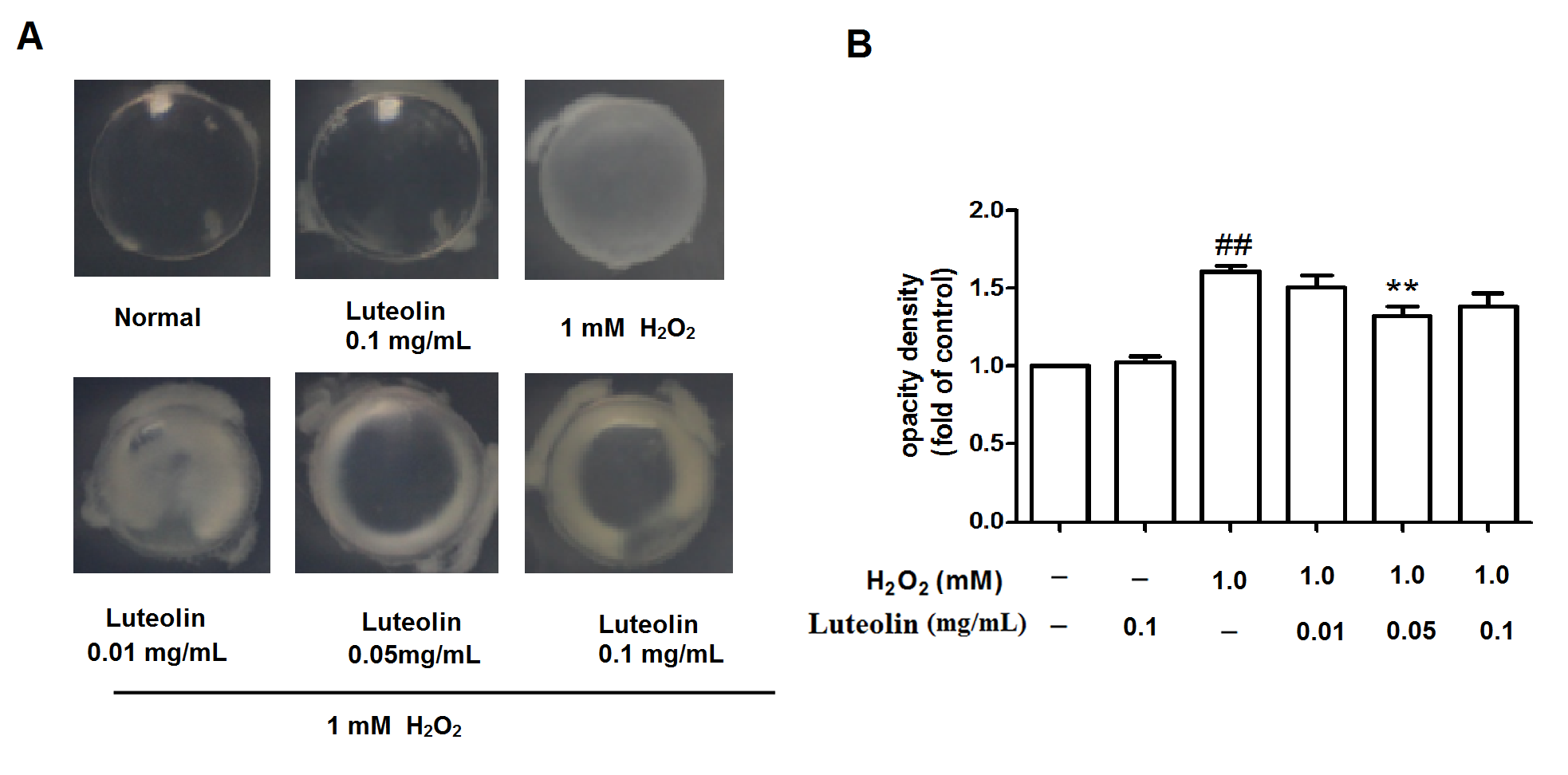

Supplement: Supplementary file 2 — Figure S1. Effects of luteolin on lens opacity in H2O2-induced cataract. Lenses were pretreated with luteolin (0.01, 0.05 and 0.1 mg/mL) for 24 h, followed by incubation with H2O2 (1 mM) for another 24 h and then recovered in fresh medium for 48 h. Data are expressed as the mean ± SEM (n = 6); ##P < 0.01, compared with the normal control group; **P < 0.01 compared with the H2O2 treatment group. (TIF 584 kb) [file 12906_2019_2555_MOESM2_ESM.tif]

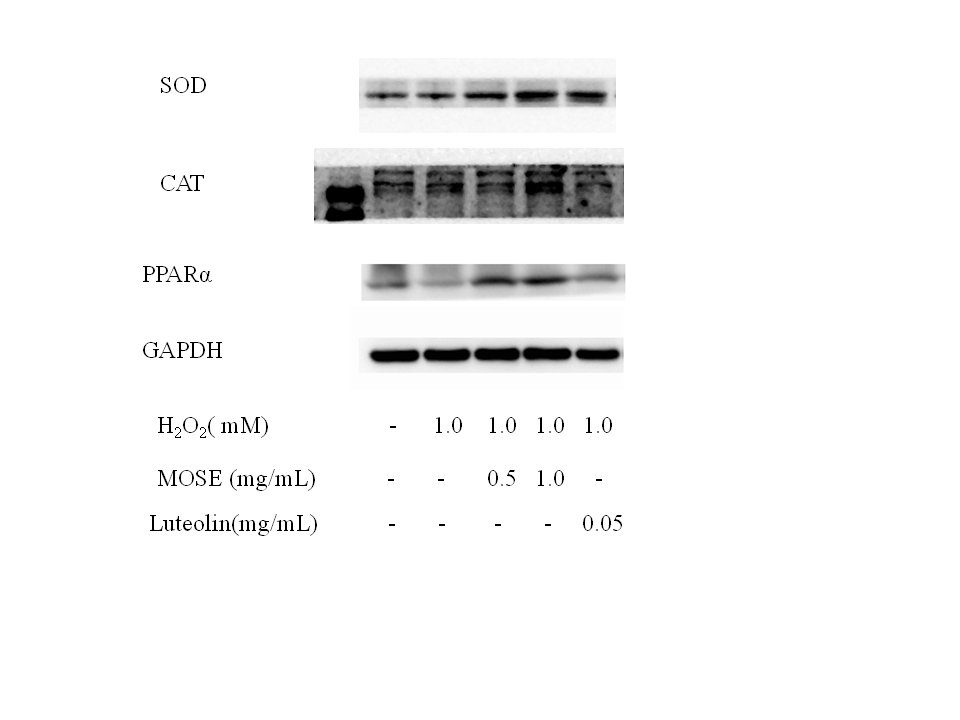

Supplement: Supplementary file 3 — Figure S2. The original gel images of Fig. 4a. (TIF 155 kb) [file 12906_2019_2555_MOESM3_ESM.tif]
